# Metal-Free Carbon Quantum Dots Implant Graphitic Carbon Nitride: Enhanced Photocatalytic Dye Wastewater Purification with Simultaneous Hydrogen Production

**DOI:** 10.3390/ijms21031052

**Published:** 2020-02-05

**Authors:** Lilei Zhang, Jingxiao Zhang, Yuanyu Xia, Menghan Xun, Hong Chen, Xianghui Liu, Xia Yin

**Affiliations:** 1 College of Chemistry and Chemical Engineering, Luoyang Normal University, Luoyang 471934, China; zhanglilei@outlook.com; 2 College of Food and Drug, Luoyang Normal University, Luoyang 471934, China; 3 School of Chemical Engineering and Pharmacy, Wuhan Institute of Technology, Wuhan 430205, China

**Keywords:** photocatalysis, wastewater purification, hydrogen production, carbon quantum dot, graphitic carbon nitride

## Abstract

The use of photocatalysts to purify wastewater and simultaneously convert solar energy into clean hydrogen energy is of considerable significance in environmental science. However, it is still a challenge due to their relatively high costs, low efficiencies, and poor stabilities. In this study, a metal-free carbon quantum dots (CQDs) modified graphitic carbon nitride photocatalyst (CCN) was synthesized by a facile method. The characterization and theoretical calculation results reveal that the incorporation of CQDs into the g-C_3_N_4_ matrix significantly improves the charge transfer and separation efficiency, exhibits a redshift of absorption edge, narrows the bandgap, and prevents the recombination of photoexcited carriers. The hydrogen production and simultaneous degradation of methylene blue (MB) or rhodamine B (RhB) in simulated wastewaters were further tested. In the simulated wastewater, the CCN catalyst showed enhanced photodegradation efficiency, accompanied with the increased hydrogen evolution rate (1291 µmol·h^−1^·g^−1^). The internal electrical field between the g-C_3_N_4_ and the CQDs is the main reason for the spatial separation of photoexcited electron-hole pairs. Overall, this work could offer a new protocol for the design of highly efficient photocatalysts for dye wastewater purification with simultaneous hydrogen production.

## 1. Introduction

The application of photocatalysts to purify wastewater with simultaneous hydrogen production has received persistent interest [1]. To efficiently convert solar energy and achieve the dual functions, for example, purifying wastewater and producing hydrogen from wastewater, the photocatalysts must satisfy the following characteristics: First, it must possess suitable bandgap which satisfies not only efficient light absorption but also suitable conduction band (CB) and valance band (VB) potentials for both wastewater purification and hydrogen production and secondly, it should own a high separation and transfer efficiency of photogenerated electrons and holes, because the recombination of photoinduced charges generally takes place at a very fast timescale (ps-µs) [2]. On the basis of the above characteristics, numerous studies have provided various types of semiconductor-based photocatalysts, such as halide, oxyhalide, metal molybdate, sulfide, metal oxide, and tungstate, in order to achieve higher photocatalytic efficiency [3,4,5]. Unfortunately, current photocatalysts still have several fundamental problems that need to be solved, such as low photoabsorption efficiency (which leads to the low quantum efficiency), high electron-hole recombination rate, high cost, and poor stability [6]. Thus, it still needs a follow-up study to solve these defects and develop an efficient photocatalyst.

Since the report of Wang and Domen documented that a polymer semiconductor based on defective graphitic carbon nitride (g-C_3_N_4_) owns the performance of hydrogen evolution from water [7], the research towards this promising nontoxic metal-free photocatalyst has attracted considerable attention, due to its facile synthesis, visible-light responding energy gap (E_g_ = 2.68 eV), high physicochemical stability, and “earth-abundant” nature [8,9]. More importantly, g-C_3_N_4_ is easily fabricated by thermal polymerization of abundant nitrogen-rich precursors such as melamine [9], dicyandiamide [10], cyanamide [11], urea [12], thiourea, and ammonium thiocyanate [13]. Moreover, this material has enormous application potential, such as organic pollutant degradation [14]. However, they still have some fatal defects restricting their practical applications, such as low quantum efficiency, high electron-hole recombination rate, low charge migration rate, high electron-hole recombination rate, the usage of noble metals as cocatalysts or reagents (such as Pt and Pd, which leads to high cost), and small surface area. For example, the quantum efficiency for hydrogen evolution is only 1%, which results in low photocatalytic activity [15,16]. Therefore, in order to enhance the application potential of g-C_3_N_4_, many strategies were developed, such as coupling with other different materials and morphology control [16,17]. Recently, carbon quantum dots (CQDs) have been successfully deposited on the g-C_3_N_4_ backbone to enhance its photocatalytic activity through increasing the electron transfer and reservoir properties [18,19,20,21]. Since then, several types of CQDs modified g-C_3_N_4_ nanocomposites have been produced and have been proven to possess an impressive performance for photocatalytic dye wastewater purification or solar water splitting. However, to the best of our knowledge, only a few reports have studied both the theoretical basis and the possible applications in dye wastewater purification with simultaneous hydrogen production [22,23,24].

In this study, one novel CQDs modified g-C_3_N_4_ nanosheets (CCN) was fabricated via a facile and environment-friendly approach. The photocatalytic performances were systematically evaluated by decomposing dye wastewater (including rhodamine B (RhB) and methylene blue (MB)) and simultaneous hydrogen production. The focus of this research was on the relationship between photocatalytic degradation of dye wastewater and hydrogen production over an as-prepared photocatalyst. The underlying mechanism of the enhanced photocatalytic properties by the CCN composite system was further deciphered by first-principles calculation, which is essential for understanding the synergistic mechanism of photocatalytic degradation with simultaneous hydrogen evolution.

## 2. Results and Discussion

### 2.1. Characterization of Photocatalysts

**Figure 1:**
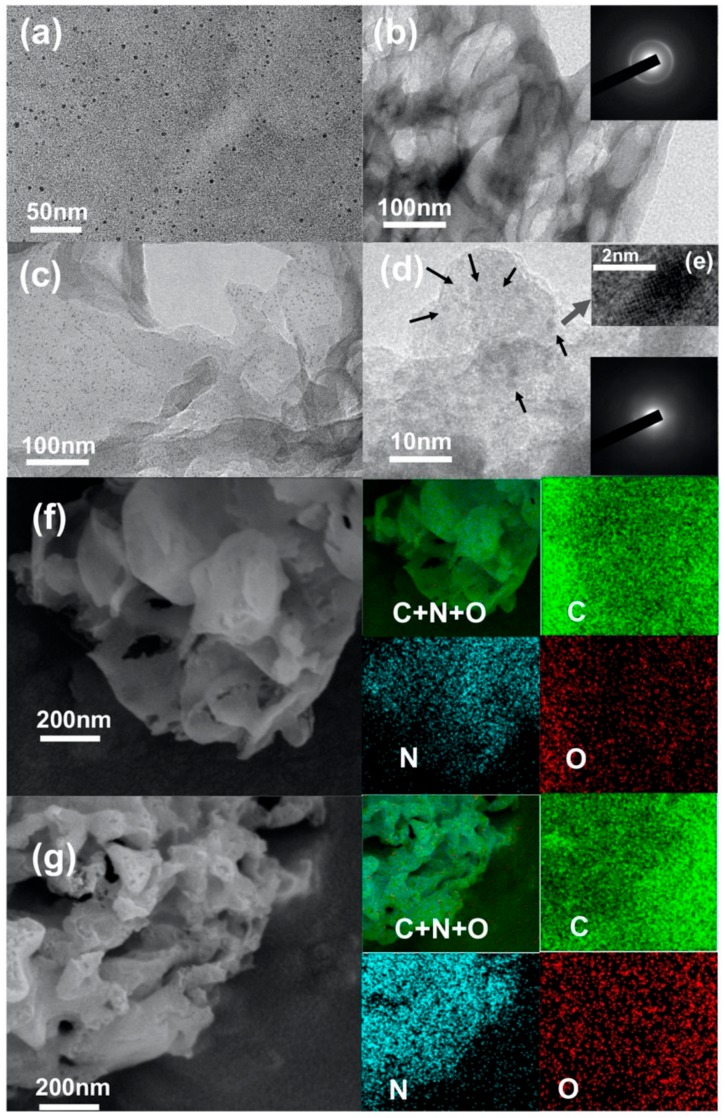
Transmission electron microscope (TEM) images of carbon quantum dots (CQDs) (**a**); g-C_3_N_4_ sheets (CN) (**b**); (**c**) low- and; (**d**) high-magnification TEM images of carbon nitride photocatalyst (CCN); (**e**) HRTEM image of a CQD embedded in CCN, and SEM images together with elemental mapping of CN (**f**); and CCN (**g**).

The TEM image of the synthesized CQDs (as displayed in Figure 1a) shows that the incomplete combustion of citric acid produces CQDs that are relatively monodisperse, mostly in diameters of 2 to 5 nm. The TG and DTG analytical results (Figure S1 in Supporting Materials) indicated that the synthesized CQDs were stable under nitrogen. From Figure 1b, a two-dimensional (2D) layered structure with chiffon-like ripples and wrinkles could be seen in the CN sample, which was in agreement with the report [15]. After modification, many particles (dark gray) with diameters of 2 to 5 nm are non-uniformly decorated on the surface of the CN (as shown in Figure 1c,e). Comparing Figure 1b with Figure 1c–e, we concluded that the particles in Figure 1c should be CQDs instead of g-C_3_N_4_ quantum dots because the reaction conditions of synthesizing CN were the same as those of CCN, except that the latter was produced by adding the CQDs as a starting material. The diffraction of the CN (inset images of Figure 1b) exhibited crystalline lobes, while those of the CCN (inset images of Figure 1d) were hazy, indicating that the matrix of the CCN composite was more disordered to the pure CN matrix. Additionally, the SEM images of the CN and CCN together with element mapping (Figure 1f,g) illustrated that the N and C elements appeared in almost the same place, indicating the CQDs were uniformly dispersed on the g-C_3_N_4_.

**Figure 2:**
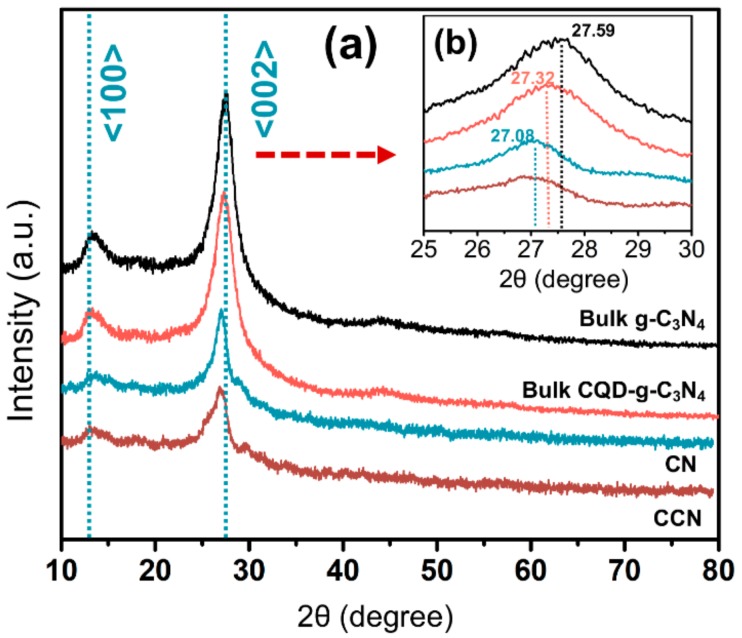
X-ray diffraction (XRD) patterns of the bulk g-C_3_N_4_, bulk CQDs-g-C_3_N_4_, CN, and CCN (**a**); and enlarged view of the 25° to 30° region (**b**).

Figure 2 shows the XRD patterns of the bulk g-C_3_N_4_, bulk CQDs-g-C_3_N_4_, CN, and CCN. According to the report [25], a stronger diffraction characteristic peak at about 27.5° and a weaker one at about 13.1° were observed from the XRD pattern of pure g-C_3_N_4_ (as displayed in Figure 2a), which corresponded to <002> and <100> crystal planes of graphitic structure. As shown in Figure 2a, the sharp XRD peaks at 27.5° corresponded to interlayer stacking of aromatic segments with a distance of 0.329 nm for g-C_3_N_4_ and 0.331 nm for CQDs-g-C_3_N_4_ from the line width analysis of the <002> diffraction peak using the Debye–Scherrer equation. This phenomenon indicates that due to CQDs modification, the spacing between layers of g-C_3_N_4_ becomes bigger. It is worth noting that the <002> peaks of the CN and CCN decreased and become smaller (Figure 2b) after the corrosion of nitric acid solution, which illustrates that the CN and CCN formed approximate two-dimensional structures.

**Figure 3:**
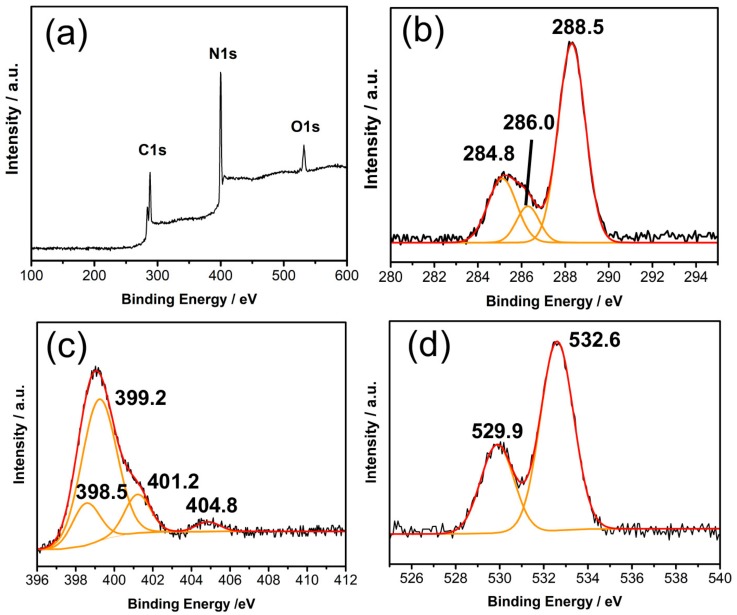
X-ray photoelectron spectroscopy (XPS) survey spectrum of 4CCN (**a**); the high-resolution XPS spectra of C 1s (**b**); N 1s (**c**); and O 1s (**d**). The black lines are the recorded XPS spectrum, while the red lines are the fitted curves of black lines, deconvoluted into the orange contributions.

Figure 3a shows the XPS spectra of 4CCN. The XPS survey spectra demonstrate that the CQDs-g-C_3_N_4_ samples are mainly composed of carbon, nitrogen, and oxygen, and their corresponding photoelectron peaks appear at binding energies of 288 (C 1s), 401 (N 1s), and 530 eV (O 1s). The high-resolution XPS spectra of C 1s (Figure 3b) is deconvoluted into three peaks centered at 284.8, 286.0, and 288.5 eV, respectively. The C peak at 288.5 eV corresponds to sp^2^ hybridized carbon (N–C = N) of g-C_3_N_4_, and the weaker one at 284.8 eV is assigned to graphitic carbon (C = C) of CQDs. The peak located at 286.0 eV is identified as a small quantity of carbon combined with C–N [26]. The N 1s spectrum (Figure 3c) displays four peaks with binding energies of 398.5, 399.2, 401.2, and 404.8 eV, respectively, which are attributed to the sp^2^-hybridized nitrogen groups (N–C = N), tertiary nitrogen atoms (N-(C)_3_), hydrogen-bonded nitrogen groups (C-N-H) and the charging effects or positive charge localization in the heterocycles, [27] respectively. Figure 3d represents the O 1 s peaks at 529.5 and 532.6 eV, which are assigned to lattice oxygen and adsorbed water, respectively. In the entire spectrum, no characteristic peak related to any other impurities is observed. On the basis of the XPS analysis, it indicates that the as-prepared CCN photocatalyst is free from other unintentional impurities. In addition, the XPS characterization showed that there was no chemical state other than CN and CQDs. Combining with TEM images, we speculated that the CQDs were deposited on the CN without covalent mixture. After acid treatment, the elemental ratio of bulk g-C_3_N_4_ was not obviously changed (Table S1 in Supporting Materials), indicating that the increase of C element content is attributed to the CQDs modification. The CQDs content was calculated by the C and N element ratio, as shown in Table S1.

### 2.2. Photocatalytic Activity

The photocatalytic activities of different photocatalysts were evaluated by the degradation of organic pollutants (RhB and MB) with the simultaneous production of hydrogen. According to the results of the preliminary experiment, the degradation profiles of RhB and MB over samples is described by the first-order equation (Equation (1))

(1) $$\ln\frac{c_{0}}{c}=kt$$

where k is the rate constant (min^−1^), c_0_ is the initial concentration of the MB or RhB solution, and c is the actual concentration of the MB or RhB solution at a specific time.

**Figure 4:**
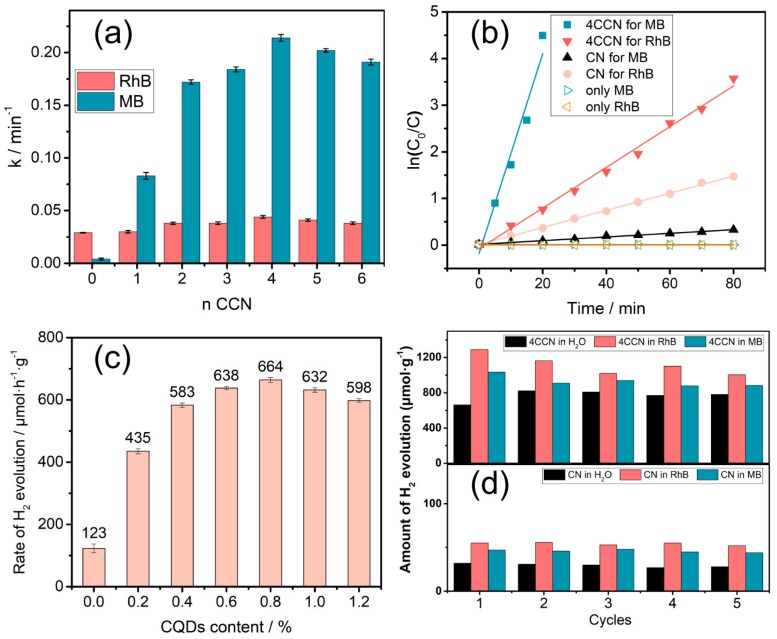
The degradation rate constant of dyes over the g-C_3_N_4_-based samples modified by different concentrations of CQDs (**a**); photocatalytic degradation profiles of RhB and MB over CN and 4CCN (0.98 wt% CQDs) (**b**); hydrogen evolution rate of the g-C_3_N_4_-based samples modified by different concentrations of CQDs (**c**); and photocatalytic hydrogen evolution per hour in different cycles over CN and 4CCN samples (**d**). Error bars represent mean ± s.d. of at least three independent experiments.

The photocatalytic rates of g-C_3_N_4_-based samples modified by different concentrations of CQDs were investigated under light irradiation, and the rate constant k was employed to analyze the catalytic efficiencies quantitatively, as shown in Figure 4a,b. First of all, without any photocatalyst, only MB or RhB did not have any change during light illumination. As shown in Figure 4a, in the presence of CN under light irradiation, RhB was found to be degraded with a high degradation rate constant of k = 0.03 min^−1^, while a low degradation rate of MB achieved (0.004 min^−1^). Subsequently, the photocatalytic degradation rates of RhB and MB over CCN both, first, increase and, then, decrease, with an increase in the amount of CQDs. It is clearly seen that 4CCN (0.98 wt% CQDs) catalyst displays the highest degradation rate constants of both MB and RhB. Therein, this catalyst shows rapid MB degradation with a rate constant of 0.214 min^−1^, which is almost 54 times higher than CN (0.004 min^−1^), while the degradation rate constant of RhB just slightly increases to 0.048 min^−1^. Thus, the as-obtained CCN photocatalyst enhances the photocatalytic activities for the degradation of MB and RhB.

The photocatalytic hydrogen production activities of CN in water with different amounts of CQDs were tested by gas chromatography under visible light irradiation, and the results are shown in Figure 4c. It was found that the 4CCN photocatalyst exhibits the highest hydrogen evolution rate (664 µmol·h^−1^·g^−1^). Therefore, in the following experiments, the optimum photocatalyst 4CCN was employed to decipher the photocatalytic process with simultaneous hydrogen production.

The photocatalytic hydrogen production activities of CN and 4CCN in polluted water solutions in different cycles were tested, as displayed in Figure 4d. A comparison of photocatalysts in water solution, shows that both CN and CCN photocatalysts in RhB and MB solution exhibit the higher hydrogen generation rates. For example, the H_2_ generation rate of 4CCN catalyst in RhB solution significantly increased (1291 μmol h^−1^·g^−1^), which was higher than that in water (664 μmol·h^−1^·g^−1^). Additionally, CN and 4CCN showed excellent stability in the process of recycling, according to Figure 4d. The results of the hydrogen evolution rate demonstrate that modified g-C_3_N_4_ by CQDs improves the H_2_ generation rate in wastewater solutions. In conclusion, after modification by CQDs, photocatalytic efficiency of the as-synthesized photocatalyst was more efficient for photocatalytic wastewater purification and energy recovery.

A comparative study of the photocatalyst was carried out with different reported photocatalysts (as displayed in Table 1). The results exhibit that CCN shows superior photocatalytic performance (including H_2_ evolution rate and dye degradation rates) as compared with the reported photocatalysts.

There are many influencing factors of the photocatalyst performance, such as specific surface area, light absorptivity, separation, and transference of electron-hole pairs, and recombination of photoexcited carriers. The specific surface test results (Table S1 in Supporting Materials) indicate that it is not an essential factor in these g-C_3_N_4_ based samples due to their close values, so other factors were explicitly investigated.

**Figure 5:**
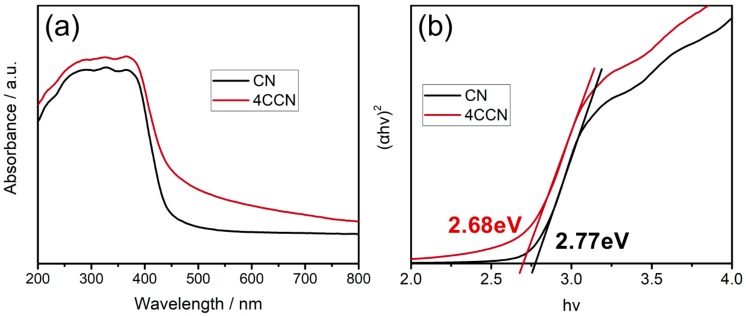
UV-Vis absorption spectra (**a**) and Tauc plot (**b**) of CN and CCN photocatalysts.

Ultraviolet-visible spectroscopy (UV-Vis) was used to determine the light absorptivity of all samples, and the results are shown in Figure 5. As displayed in Figure 5a, the incorporation of CQDs into the g-C_3_N_4_ matrix leads to an obvious increase in the UV-Vis absorption over the entire wavelength range investigated. The phenomenon indicates that the coupling of CQDs and g-C_3_N_4_ is beneficial to improve the light absorption of CN, which implies the enhanced photocatalytic activity.

The bandgap energy (Eg) of CN and 4CCN (Figure 2b) is estimated from the Tauc plot method based on Equation (2):

(2) $$E_{g}=\frac{{\left(\alpha hv\right)}^{r}}{hv}$$

where α, *h*, and *ν* are the absorption coefficient, Planck constant, and light frequency, respectively. The exponent r is 2 for a direct band gap material and 1/2 for an indirect bandgap material.

As shown in Figure 5b, an excellent linear fit was obtained for both 4CCN and CN when r = 2, which corresponding with the reports in the literature in which 4CCN and CN are direct bandgap materials [20,31]. By measuring the x-axis intercept of an extrapolated line from the linear region of the curve, the Eg value of 4CCN is, thus, determined to be 2.68 eV, which is 0.09 eV smaller than that of pure g-C_3_N_4_. This result reveals the fact that the CCN absorbs and utilizes visible light with a larger wavelength, which helps improve the photocatalytic efficiency.

The photoluminescence (PL) emission spectra were performed to study the transfer behavior of charge carriers, with an excitation wavelength of 350 nm. The CN has a prominent peak at 453 nm in the PL spectrum, which corresponds to the bandgap recombination of electron-hole pairs. The CCNs have longer excitation wavelength than that of the CN, due to its narrower band gap. Additionally, the intensities of PL spectra for CCN decrease with CQDs content increasing, which indicated that the recombination of charge carriers was prevented effectively in the composite samples. However, when the content of CQDs increases to a certain extent, such as 4CCN, 5CCN, and 6CCN, the inhibition of photoelectron recombination no longer increased obviously.

**Figure 6:**
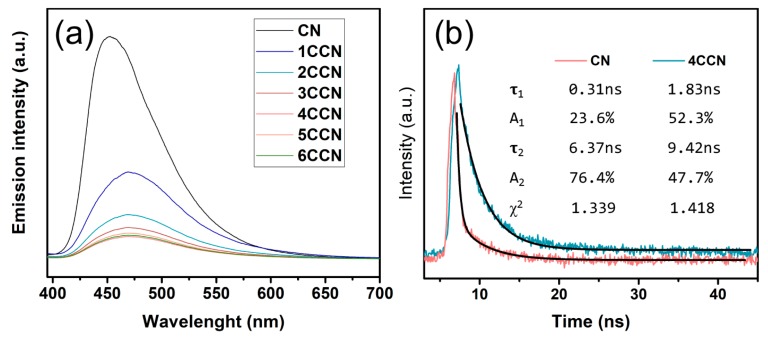
Photoluminescence spectra (**a**) of CN and CCN samples, and time-resolved photoluminescence spectra (TRPL) (**b**) of the CN and 4CCN samples.

The nanosecond level TRPL was recorded to investigate the transfer dynamics of the carriers in the CN and 4CCN under irradiation. As shown in Figure 6b, It can be seen that the lifetimes of the photoexcited charge carriers on 4CCN are prolonged compared with CN, confirming more excellent photogenerated carriers separation. It indicated that the formation of CQD-g-C_3_N_4_ heterostructures remarkably promoted charge transfer efficiency, thereby, favoring the photoexcited electron-hole pairs spatial separation and the photocatalytic reactions for dye degradation and H_2_ evolution.

**Figure 7:**
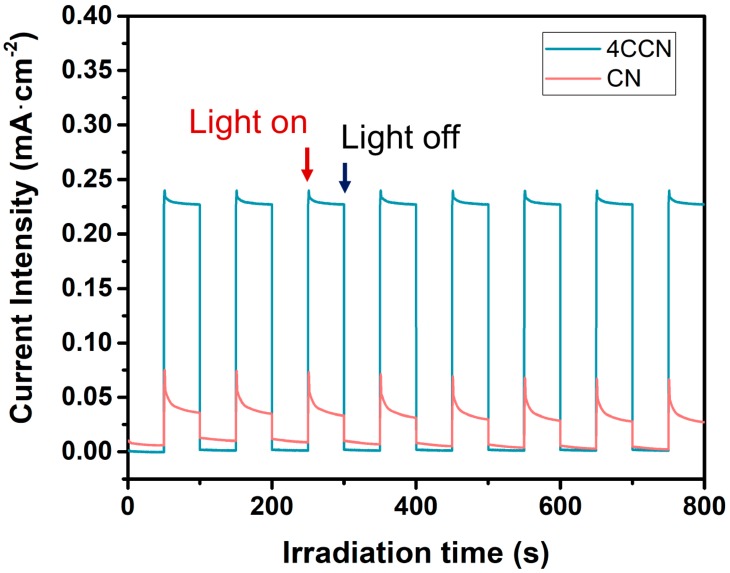
Transient photocurrent responses curves for CN and 4CCN samples under visible light irradiation.

Figure 7 shows the transient photocurrent response curves of CN and 4CCN under intermittently visible light irradiation (λ > 420 nm). The 4CCN sample shows higher photocurrent density, indicating the efficient photogenerated charge transfer between CQDs and g-C_3_N_4_. Additionally, the photocurrent spike, upon visible light irradiation, can be attributed to the recombination processes on the surface of samples. The photocurrent increases rapidly when the light turns on and, then, the holes accumulate at the photocatalysts’ surface competitively recombine with electrons from the conduction of CN, leading to decay of the photocurrent in a short time. After the equilibration of competitive separation and recombination of carriers, the photocurrent reached a constant value. The photoluminescence and photoelectrochemical results confirmed the superior charge transfer and recombination inhibition in the 4CCN composite photocatalyst.

### 2.3. Theoretical Calculation

**Figure 8:**
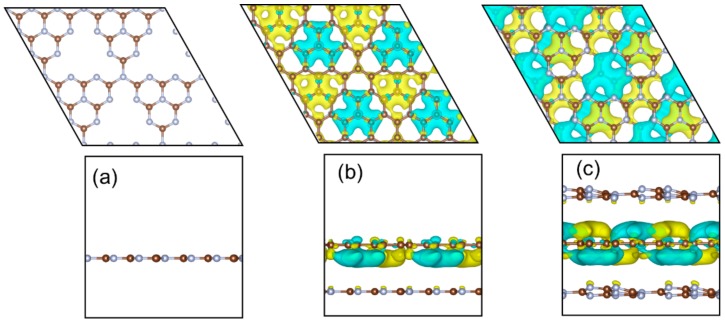
The optimization structures and different charge density of CN (**a**) and CCN (**b** and **c**), yellow (gain electron), cyan (lose electron).

DFT theoretical calculation was performed to illustrate the fundamental reason for the enhanced photocatalytic activity. According to the TEM and XPS characterization, there was no covalent structure between CQDs and g-C_3_N_4_. In order to examine the local electronic structure information of the stacked structure of CQDs and g-C_3_N_4_, a single 2D sheet (Figure 8b) was modeled to represent the CCN structure. Figure 8a,b shows the optimized structures of CN and CCN. It can be seen that the structure of g-C_3_N_4_ has been wrinkled due to the interaction between CQDs and g-C_3_N_4_. Importantly, on the CQDs surface, the charge was distributed to different regions, the charge accumulated in the region which was facing the cavities in g-C_3_N_4_, and the charge depleted in the region which was facing the heptazine unit. When g-C_3_N_4_ was excited by photoirradiation, the photoexcited carriers tended to accumulate on CQDs that were facing the cavities of g-C_3_N_4_ under the action of the internal electric field, which lead to the spatial separation of photoexcited electrons and holes. This was the main reason for the recombination prohibition of the photogenerated carriers on the CCN photocatalyst.

Additionally, it was possible to form the structure of CQDs embedded between g-C_3_N_4_ layers, and it would change the local electronic structure of CCN. Therefore, the model CQDs sheet between two g-C_3_N_4_ sheets was constructed, and Figure 8c shows the optimal structure and different charge density. It can be seen that the charge also depletes or accumulates in different regions on CQDs due to facing cavities or heptazine unit when CQDs insert into two monolayer g-C_3_N_4_.

**Figure 9:**
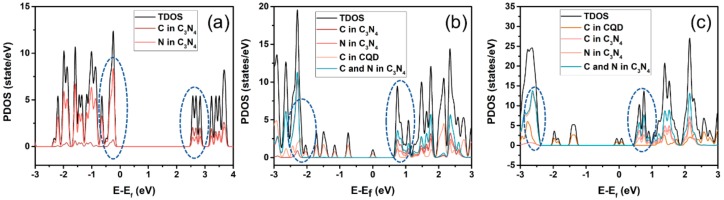
The projected density-of-states for CN (**a**) and CCN (**b** and **c**).

According to the frontier molecular orbital theory, the region near the Fermi level is critical for photocatalysis, therefore, some regions were broken out to study the hybridization between CQDs and g-C_3_N_4_, as shown in Figure 9. In the region of the dotted circle of CCN (Figure 9b,c), the peaks of C in CQDs and g-C_3_N_4_ almost appear at the same energy level, indicating that there is strong hybridization between CQDs and g-C_3_N_4_. The interaction between CQDs and g-C_3_N_4_ could be the reason for the internal electric field between CQDs and g-C_3_N_4_, which benefit for the spatial separation of photoexcited carriers.

### 2.4. The Mechanism of the Enhanced Photocatalytic Activity

**Figure 10:**
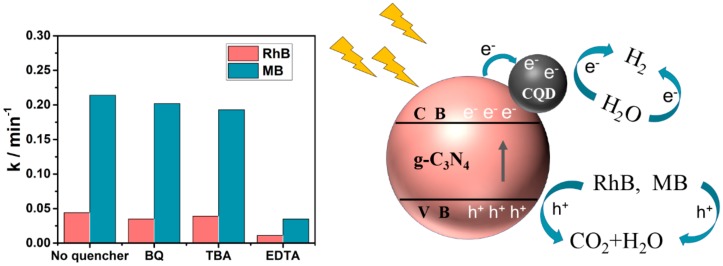
Proposed mechanism for methylene blue (MB) and RhB degradation by CCN under light irradiation.

The primary reactive species for the photocatalytic oxidation contains superoxide radicals (·O_2_^₋^), photogenerated holes (h^+^), and hydroxyl radicals(·OH). In order to further understand the reaction mechanisms, three scavengers were used to explore the reactive species in photocatalytic degradation. The 5 × 10^₋3^ mol/L solutions of tertiary butanol (TBA), p-benzoquinone (BQ), and ethylenediaminetetraacetic acid disodium (EDTA) were employed as scavengers for ·OH, O_2_^₋^ and h^+^, respectively. As shown in Figure 10, the photocatalytic degradation rate of RhB and MB degradation over 4CCN was almost invariable with the addition of BQ and TBA. However, the photocatalytic activity decreased with the addition of EDTA, indicating that the photocatalytic process was mainly governed by h^+^. It is well-established that efficient separation of photoexcited electrons and holes is of great importance for improving the photocatalytic activity.

On the basis of the characterizations mentioned above and the DFT theoretical calculation results, the possible mechanism of charge transfer in CCN was proposed to dissect the enhanced photocatalytic activity, as shown in Figure 10. Under the influence of the internal electric field formed between g-C_3_N_4_ and CQDs, the photogenerated electrons easily transferred to the CQDs, and the photogenerated holes remained on the g-C_3_N_4_, thereby forming a spatial separation. Then, the h^+^ was used for the oxidative decomposition of the dye, at the same time, the photogenerated holes were used to reduce H^+^ to form H_2_, thereby achieving efficient decomposition of the dye and simultaneous hydrogen production.

## 3. Materials and Method

### 3.1. Reagents and Materials

All reagents (analytical purity) in the experiment were obtained from Sinopharm Chemical Reagent Co., Ltd. (Shanghai, China) and used without further purification. The ultrapure water used in all experiments was from a synergy ultrapure water system (Millipore).

### 3.2. Synthesis of Photocatalysts

A simple bottom-up approach was used to prepare CQDs. First, 1g of citric acid was heated to 180 °C for 20 min. During the heating process, the color gradually changed from white to brown, and a large number of bubbles were generated. Subsequently, a NaOH solution (0.5 mol·L^−1^) was slowly added until neutral, and the mixed solution was subjected to an ultrasonic treatment for 20 min. Then, the mixture was centrifuged at 8000 rpm for 10 min. After that, the supernatant was dialyzed in a dialysis bag (1 KDa molar mass) for two days to remove the salt.

The method for the preparation of g-C_3_N_4_ sheets (CN) was comprised of the following steps: First, 10 g of urea was placed in an alumina crucible with a cover and, then, heated to 550 ℃ with a heating ramp of 2 °C·min^−1^ and maintained at this temperature for two hours. After calcination treatment, the obtained power was light yellow, implying the formation of bulk g-C_3_N_4_. Then, 100 mg bulk g-C_3_N_4_ were stripped in 40 mL HNO_3_ solution (V_HNO_3__/V_H_2___O_ = 1:1) at 80 °C for 1 hour. Then the obtained mixture was diluted with deionized water and adjusted to neutrality with sodium hydroxide. Subsequently, the CN was collected by centrifugation at 8000 rpm for 5 min and washed three times with water for removal of salt.

CCN was prepared as follows: The mixture of 10 g urea with different volume of CQDs solution was heated to 550 °C at a heating rate of 2 °C·min^−1^ under N_2_ and maintained at this temperature for two hours. Then, 100 mg obtained powder (bulk CQDs-g-C_3_N_4_) was stripped in the HNO_3_ solution (40 mL, V_HNO_3__/V_H_2___O_ = 1:1) at 80 °C for 1 h. Then the obtained mixture was diluted with deionized water (50 mL) and adjusted to neutrality with sodium hydroxide in the ice-water bath. Subsequently, the CCN was collected by centrifugation at 8000 rpm for 5 min and washed three times with water for removal of salt. The CQDs content was analyzed by flash 2000 Elemental analyzer, as shown in Supporting Materials.

### 3.3. Photocatalytic Reaction

Photocatalytic dyeing wastewater purification and H_2_ production were investigated under BL-GHX-V photochemical reactor (BILON Corporation, China). Rhodamine B (RhB) and methylene blue (MB) were employed as the probe molecules. Before light irradiation, 10 mg of as-prepared sample was added into 30 mL of dye solution (15 mg·L^−1^) and, then, the suspension was stirred for 30 min to achieve the adsorption-desorption equilibrium in the dark. A 300 W Xenon lamp (320 to 780 nm) was used as a light source, which was positioned 10 cm away from the reactor with an average light intensity of 168 mW·cm^−2^. After irradiation, the suspension was taken out and centrifuged to remove the photocatalysts before measurement. The concentration changes of RhB and MB were monitored by measuring the UV-Vis absorption of the liquid supernatants. In addition, the formed hydrogen was analyzed by gas chromatography (GC7900, Techcomp) with a thermal conductivity detector (TCD) and a 5Å molecular sieve capillary column. The carrier gas was high purity nitrogen. The detector response was calibrated by the standard mixed gas of H_2_ and N_2_, which was supplied by Henan Yuanzheng corporation.

### 3.4. Characterization

All of the phase compositions and crystal structures of the prepared samples were determined by powder X-ray diffraction (XRD) method using BRUKER D8-ADVANCE diffractometer with Cu Kα radiation (2θ = 10°∼80°, λ = 0.15418 nm). The morphology of the as-prepared samples was observed by a scanning electron microscopy (SEM, Zeiss Sigma 500) and a transmission electron microscope (TEM, JEOL2010), respectively. UV-Vis diffuse reflectance spectra (DRS) of the samples were measured by Shimadzu UV-3600P UV-Vis spectrophotometer. X-ray photoelectron spectroscopy (XPS) (PHA-5400, Al Kα radiation) was employed to analyze the chemical composition. The binding energy was calibrated using the C 1s peak (BE = 284.8 eV) as standard. The photoluminescence (PL) spectra of the samples were performed on a Hitachi F-7000 spectrofluorometer equipped with a 450 W Xenon lamp. Thermogravimetric analysis of CQDs was performed by Diamond TG/DTA6300 comprehensive thermal analyzer under nitrogen. Time-resolved photoluminescence spectra (TRPL) was recorded on an FLS1000 Edinburgh analytical instrument apparatus. With a three-electrode system and 0.5 mol·L^−1^ Na_2_SO_4_ aqueous solution as the electrolyte, the photocurrent response of the samples was tested by a CHI 660E electrochemical station (Chenhua, China). The Ag/AgCl electrode and platinum wire were reference electrode and counter electrode, respectively. The glassy carbon electrodes containing the as-prepared products were served as the working electrodes.

## 4. Conclusions

In summary, we developed a facile method to prepare a novel carbon quantum dot modified g-C_3_N_4_ (CCN), which was used to purify dye wastewaters (MB and RhB) with simultaneous hydrogen production. As tested for photocatalytic activities under visible light, CCN displayed a significant enhancement in both MB and RhB degradation and hydrogen production efficiencies. It is worth noting that the 4CCN entirely degraded MB within 20 mins. The characterization and DFT theoretical calculation results indicated that the incorporation of CQDs into the g-C_3_N_4_ matrix significantly improves the charge transfer and separation efficiency of the e^₋^/h^+^ pairs under the internal potential fields between CQDs and g-C_3_N_4_. A proposed mechanism speculated that the photogenerated electrons of g-C_3_N_4_ could migrate to CQDs quickly for the reduction of H^+^ to H_2_, and the photogenerated holes would stayed on g-C_3_N_4_ for simultaneous oxidation of dyes, which would provide new lines of evidence for the wastewater purification with simultaneous energy production.

## Figures and Tables

**Table 1:** Comparative photocatalytic results of the present work with reported catalysts for H_2_ evolution rate and dye degradation rates.

Photocatalyst	Light Source	Catalyst Dose (g/L)	H_2_ Evolution Rate	Dye Degradation	Reference
CQDs/C_3_N_4_	AM 1.5 solar power system with a cutoff filter (λ > 420 nm)	0.4	152 µmol·g^₋1^·h^₋1^ in 10 mg·L^₋1^ bisphenol A solution		[18]
BBT/TiO_2_	300 W Xe lamp with a 420 nm cut-off filter	1	∼5933 µmol·g^₋1^·h^₋1^ with Pt as cocatalyst and TEOA as sacrificial reagent		[22]
5MoS_2_QDs@ZnIn_2_S_4_@RGO1	300 W xenon arc lamp (320–780 nm)	1.25	47.22 µmol·g^₋1^·h^₋1^ in RhB degradation; 0 μmol/h/g in MB degradation	Degradation of MB: 98.5% after 12 h; Degradation of RhB: 98.8% after 12 h	[23]
CDs/CdS/GCN	300 W Xe lamp with a cutoff filter (λ > 420 nm)	0.63	3.4 µmol·g^₋1^·h^₋1^ in pure water		[24]
C-dots/g-C_3_N_4_/TiO_2_	350 W Xe arc lamp	0.63	205 µmol·g^−1^·h^−1^ in triethanolamine aqueous solutions		[28]
CQDs/Bi_2_MoO_6_	300 W Xe lamp with a cutoff filter (λ > 420 nm)	1		Degradation of MB: 100% after 120 min; Degradation of RhB: 100% after 120 min	[29]
CQDs/Ag/Ag_2_O	150 W infrared lamp (λ > 700 nm)	0.5		Degradation of MB: 37% after 150 min; Degradation of RhB: 48% after 150 min	[30]
CCN	300 W Xenon lamp (320–780 nm)	0.33	1291 µmol·g^₋1^·h^₋1^ in RhB degradation	Degradation of MB: 100% after 20 min; Degradation of RhB: 100% after 110 min	Present Work

## Supplementary Materials

Supplementary materials can be found at https://www.mdpi.com/1422-0067/21/3/1052/s1.
