# Supplementary material for: Metal-Free Carbon Quantum Dots Implant Graphitic Carbon Nitride: Enhanced Photocatalytic Dye Wastewater Purification with Simultaneous Hydrogen Production
Source: Int J Mol Sci. 2020 Feb 5;21(3):1052. doi: 10.3390/ijms21031052 (PMC7036787; doi:10.3390/ijms21031052)
Supplement: Supplementary file 1 [file ijms-21-01052-s001.pdf]

Supporting Information

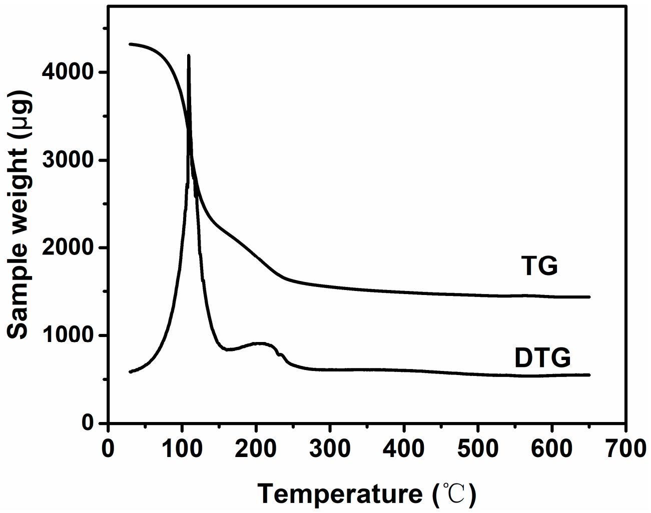

Figure S1. TG and DTG profiles of the synthesized CQDs at heating rate of 15 °C/min under Nitrogen. The two apparent weightless peaks at 110 °C and 200 °C were attributed to the volatilization of the solvent adsorbed on the CQD surface. No visible weightless peak was found above 400 °C, indicating that pure CQDs were stable under the experimental conditions.

Table S1 CN and CCN CQD content and BET surface

CCN	C:N ratio	CQD contents	BET surface m2/g
Bulk g-C_3N_4	0.75	0	73.4
CN	0.75	0	92.8
1CCN	0.753	0.16	88.0
2CCN	0.757	0.36	83.7
3CCN	0.765	0.78	77.7
4CCN	0.769	0.98	87.1
5CCN	0.776	1.34	84.8
6CCN	0.783	1.69	82.5

$$CQD\ content = \frac{12 \times (CN\ ratio - 0.75)}{12 \times CN\ ratio + 14}$$
